# Supplementary material for: Remodeling and self-healing of individual amyloid tactoids via multiphoton absorption
Source: Nat Commun. 2025 Dec 2;17:254. doi: 10.1038/s41467-025-66954-8 (PMC12783262; doi:10.1038/s41467-025-66954-8)
Supplement: Supplementary file 1 — Supplementary Information [file 41467_2025_66954_MOESM1_ESM.pdf]

Supplementary Information

Remodeling and Self-healing of Individual Amyloid Tactoids via Multiphoton Absorption

Dongdong Lin [1,2,3*], Hamed Almohammadi [1,4], Yufen Zhao [5] and Raffaele Mezzenga [1,6*]

1. ETH Zurich, Department of Health Sciences & Technology, Schmelzbergstrasse 9, Zurich 8092, Switzerland

2. Institute of Fundamental Physics and Quantum Technology, Ningbo University, Ningbo, Zhejiang 315211, P. R. China

3. School of Physical Science and Technology, Ningbo University, 818 Fenghua Road, Ningbo 315211, P. R. China

4. John A. Paulson School of Engineering and Applied Sciences, Harvard University, Cambridge, MA, U.S.A.

5. Qian Xuesen Collaborative Research Center of Astrochemistry and Space Life Sciences, Ningbo University, 818 Fenghua Road, Ningbo 315211, P. R. China

6. ETH Zurich, Department of Materials, Zurich 8093, Switzerland

***Corresponding authors:**

lindongdong@nbu.edu.cn

raffaele.mezzenga@hest.ethz.ch

Table of Contents

Supplementary Fig. 1 | Properties of amyloid fibrils. 4

Supplementary Fig. 2 | LLCPS behaviors of amyloid fibrils and the volume-composition diagram. 5

Supplementary Fig. 3 | Three types of amyloid tactoids through LLCPS. 6

Supplementary Note 1 | The theoretical calculations of three-photon absorption (3PA). 7

Supplementary Fig. 4 | β-sheet-rich amyloid fibrils. 8

Supplementary Fig. 6 | The schematic and experimental data showing bipolar and cholesteric tactoids. 10

Supplementary Note 2 | The volume calculation of tactoids. 11

Supplementary Fig. 7 | The elimination and cutting of amyloid tactoids by *3D-Heating*. 12

Supplementary Table 1 | The dynamics of half pitch values from the remaining half of the cholesteric tactoids. 13

Supplementary Table 2 | The dynamics of half pitch values from the original tactoid to two new cholesteric tactoids. 13

Supplementary Fig. 8 | The break-up of bipolar amyloid tactoids by 3D-Heating. 14

Supplementary Fig. 9 | The break-up of cholesteric amyloid tactoids by 3D-Heating and recovery of tactoid. 15

Supplementary Fig. 10 | Exposure of CNCs tactoids. 16

Supplementary Fig. 11 | The recovery rate of the disordered area. 17

Supplementary Fig. 12 | The exposure of cholesteric tactoids with patterns. 18

Supplementary Fig. 13 | Laser-induced negative tactoid and its recovery. 19

Supplementary Fig. 14 | The exposure of cholesteric tactoids with spots array and their recovery. 20

Supplementary Fig. 15 | The erasure of half cholesteric tactoids by 3D-Heating and their recovery. 21

Supplementary Table 3 | The dynamics of half pitch values from the remaining half of the cholesteric tactoids. 21

Supplementary Fig. 16 | 3D view of nanoparticle hybrid tactoids by fluorescence microscope. 22

Supplementary Fig. 17 | Laser digging arrays on a single nanoparticle hybrid tactoid and its recovery. 23

Supplementary Fig. 18 | Cutting of a tactoid into 4 pieces and its quick recovery processes. 24

Supplementary Fig. 19 | The dynamics of recovery processes of the three pieces of a cholesteric tactoid. 25

Supplementary Fig. 20. | Cutting of a tactoid along 2*R* and its recovery process. 26
Supplementary Fig. 21 | The dynamics of 2*R* and *H* of separated tactoid along *H*. 27
Supplementary Fig. 22 | Random cutting of a tactoid into pieces and its recovery processes. ...28
Supplementary Fig. 23 | High resolution cutting by 3D-Heating. 29
Supplementary Fig. 24 | Engineering of cholesteric tactoid. 30
Supplementary Fig. 25 | The imaging of a rotated bipolar tactoid. 31
Supplementary Fig. 26 | Erasing a tactoid layer for smart optical switch. 32
References 33

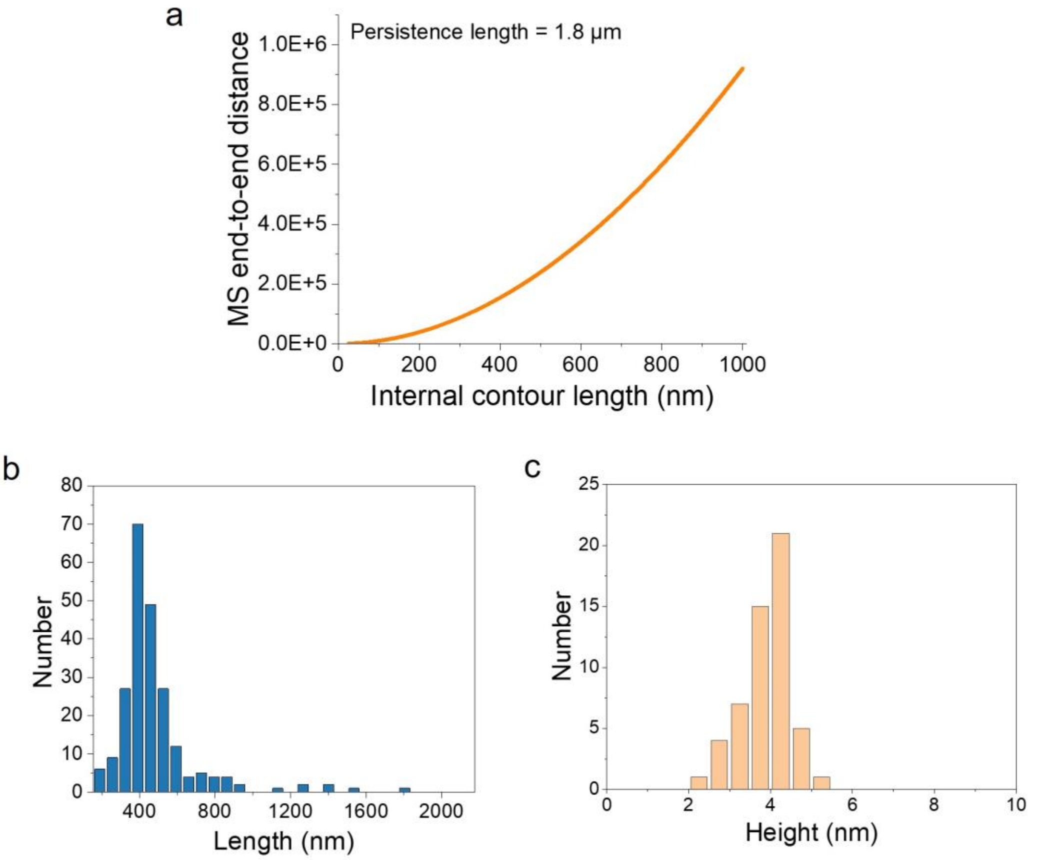

Supplementary Fig. 1 | Properties of amyloid fibrils. **a**. Plots of measured mean-square end-to-end distance vs. internal contour length from the original fibrils. The persistence length was obtained from the 2D worm-like chain model fitting[1]. **b-c**. The length and height distribution of short BLG fibrils, respectively.

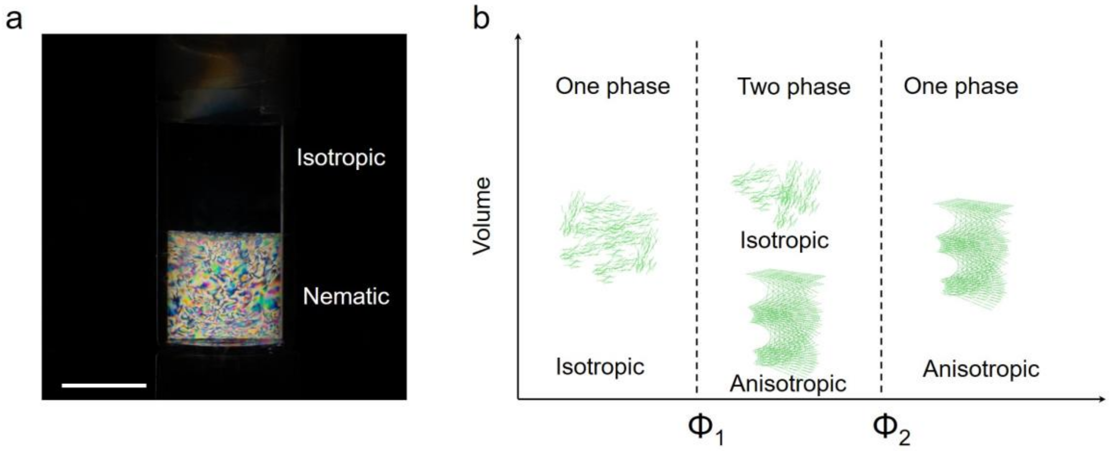

Supplementary Fig. 2 | LLCPS behaviors of amyloid fibrils and the volume-composition diagram. **a**. A suspension of amyloid fibrils at concentration in the isotropic + nematic coexistence region, captured with a crossed polarizer camera. The scale bar is 1 cm. **b**. The schematic of the volume-composition diagram with two vertical binodal lines at Onsager volume fractions Φ_1 and Φ_2.

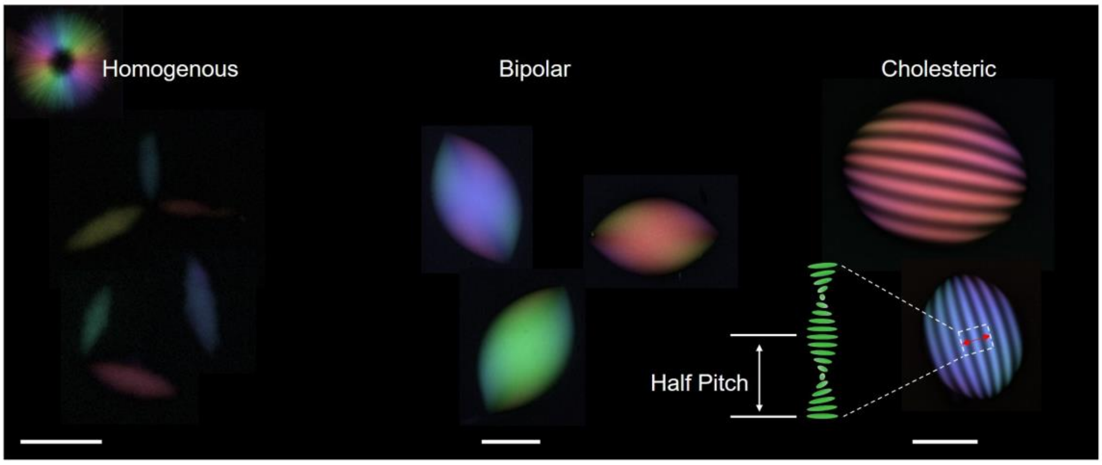

Supplementary Fig. 3 | Three types of amyloid tactoids through LLCPS. LC-PolScope images of the three classes of homogenous, bipolar, and cholesteric tactoids. The color wheel shows the direction of the director field within tactoids. The tactoids are classified based on their director field configurations. In homogenous tactoids, the director field is aligned to the long axis of the tactoid. In bipolar tactoids, the director field always follows tactoids interfaces. The uniaxial cholesteric configuration is characterized by its typical band texture and quantified by half pitch value, which is the band-to-band distance where mesogens rotate 180° along a short axis of tactoids. Scale bar is 25 µm.

Supplementary Note 1 | The theoretical calculations of three-photon absorption (3PA).

The theoretical calculations of three-photon absorption (3PA) are well documented previously[2-4]: The normalized transmittance of the open aperture Z-scan for 3 photon absorption (n=2) can be written as:

$$T(x, \Psi_n) = sinh^{-1}(\psi_n)/(\psi_n)$$

where $x = z/z_R$; z is the distance from the focus point; z_R is the Rayleigh range of the beam; $\psi_n = \Psi_n/(1+x^2)$; and Ψ_n is the peak phase shift that:

$$\Psi_n = (n\beta_n I_0 L_{eff}^{(n)})^{1/n}$$

where I_0 is the optical intensity of the beam at the focus; and β_n is the n+1 photon absorption. $L_{eff}^{(n)}$ is the effective sample length given an n+1 photon absorption process:

$$L_{eff}^{(n)} = [1 - \exp(-n\alpha_0 L)]/n\alpha_0$$

where α_0 is the linear absorption; and L is the sample length.

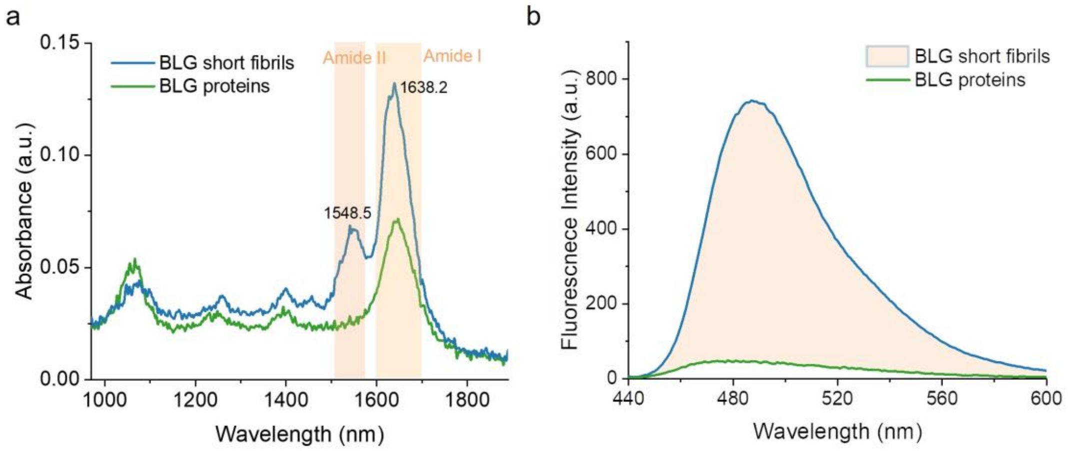

Supplementary Fig. 4 | β-sheet-rich amyloid fibrils. **a**. Fourier Transform infrared spectra of BLG proteins and BLG shortened fibrils. The amide I region refers to a specific part of the infrared (IR) spectrum, typically found between 1600 and 1700 cm^{-1}, that is primarily associated with the C=O (carbonyl) stretching vibration of the peptide bond in proteins. The amide I band is very sensitive to protein secondary structures; we can find large peaks of β-sheet: ~1612–1640 cm^{-1}. Amide II region, wavenumber range: ~1510–1580 cm^{-1}, supporting amide I data in secondary structure analysis. **b**. Thioflavin T (ThT) fluorescence spectra of BLG proteins and BLG shortened fibrils.

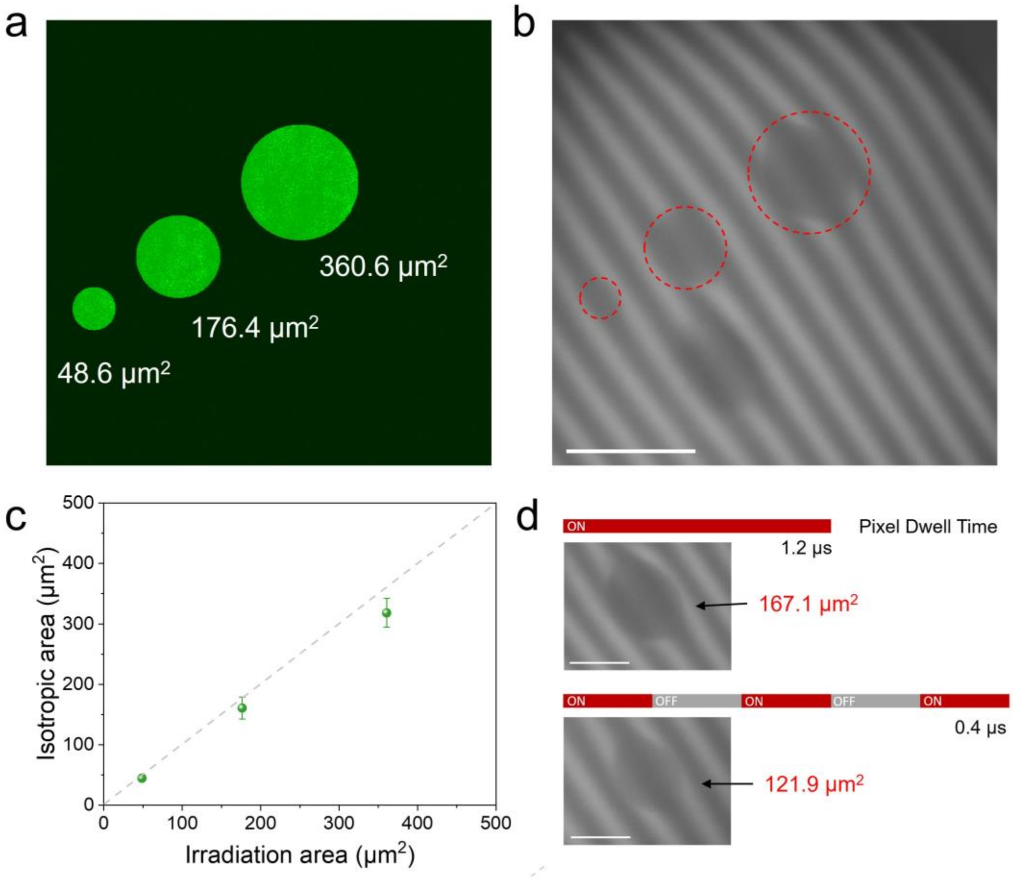

Supplementary Fig. 5 | The effects of irradiation area and time on samples of ordered amyloid fibrils. **a.** Three different irradiation areas were designed. **b.** The ensued disordered regions within the sample as a consequence of irradiation are marked with red dashed circles. Scale bar is 30 μm. **c.** The irradiation area and the resulting disordered region (isotropic area) measured within the samples are of comparable size. The data were expressed as mean ± standard deviation (S.D). **d.** The comparison between a sample irradiated with a pixel dwell time of 1.2 μs and a sample irradiated with the same total exposure time divided into three times with recovery intervals. Scale bar is 10 μm.

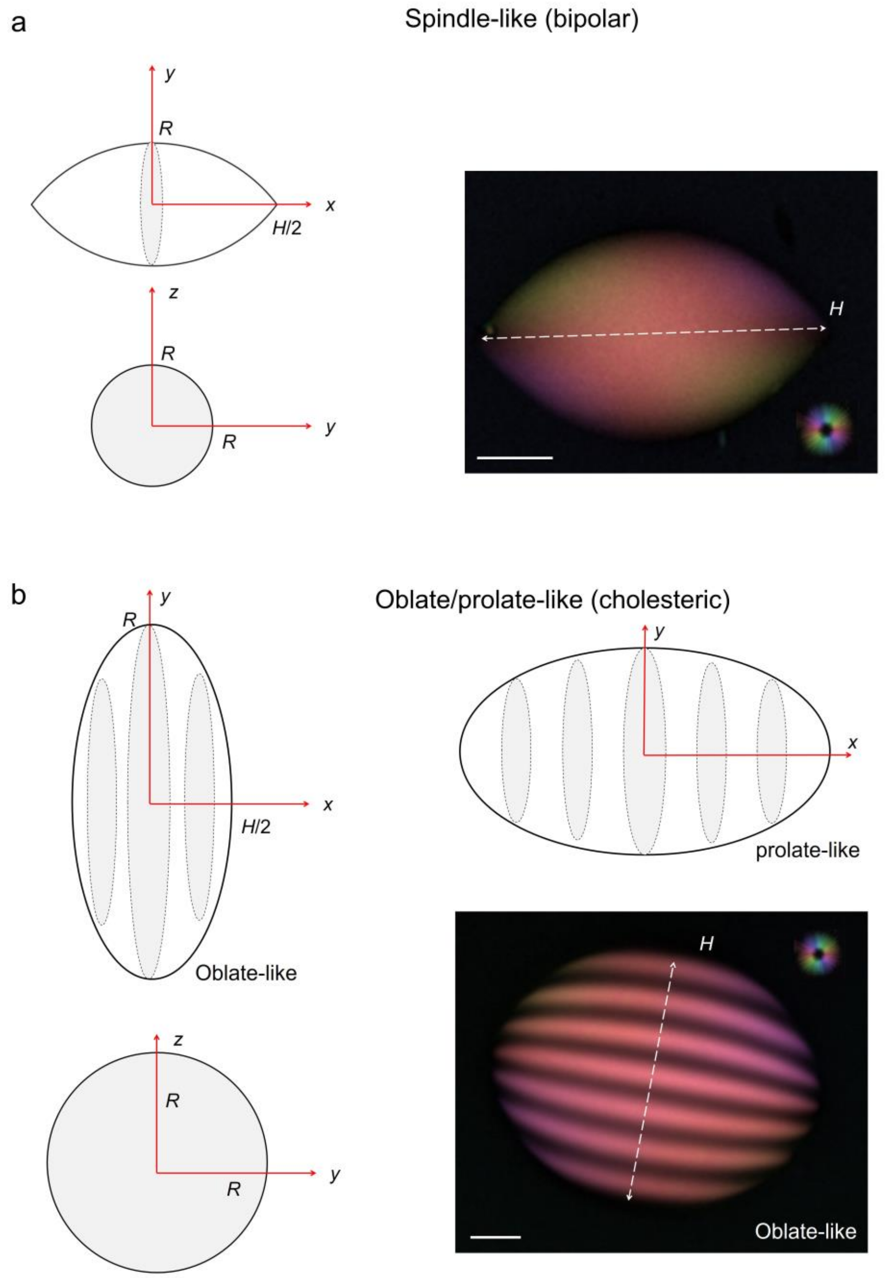

Supplementary Fig. 6 | The schematic and experimental data showing bipolar and cholesteric tactoids. **a**. Spindle-like bipolar tactoid. We define the height (H) along the long x axis, while the radius (R) in the yz plane. Here, $H > 2R$. The right panel shows a bipolar tactoid captured by the LC Pol-Scope device. The colors represent the orientation of the director field, following the color map shown in the bottom-right corner of the image. **b**. The structure of oblate/prolate-like tactoid that exhibits $H < 2R$(oblate-like) and $H > 2R$(prolate-like). The bottom-right panel shows a cholesteric tactoid (oblate-like) obtained by the LC Pol-Scope device. Scale bars are 10 μm.

Supplementary Note 2 | The volume calculation of tactoids.

The volume calculation in spindle-like amyloid fibrils tactoids[5] is as follows:

$$V = \pi\left[HR^2 - RH^2 + \frac{5}{12}H^3 + \frac{\pi}{4}\left(RH^2 - \frac{1}{2}H^3\right)\right]$$

The ellipse volume formula was used in the cholesteric tactoids (axisymmetric) to calculate the volume:

$$V = 2\pi R^2 H/3$$

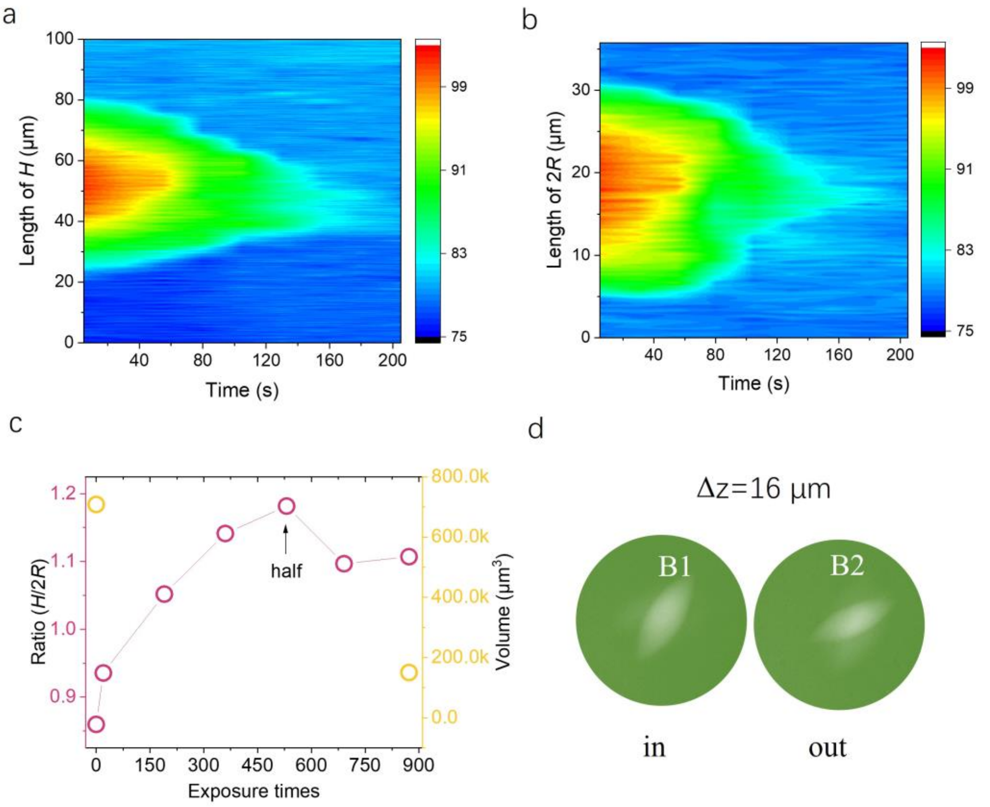

Supplementary Fig. 7 | The elimination and cutting of amyloid tactoids by *3D-Heating*. **a-b**. Dynamics of H and 2R from the POM intensity map during the exposure of bipolar tactoid in Fig. 2a, respectively. The color bars show the intensity of the POM. **c**. The changes in aspect ratio and volume of the cholesteric tactoid in main Fig. 2g, respectively. **d**. Imaging of separated bipolar tactoids B1 and B2. Their center-to-center distance was 16 µm. All of the images were captured under a crossed polarizer.

Supplementary Table 1 | The dynamics of half pitch values from the remaining half of the cholesteric tactoids.

**No.**	**0 s**	**20 s**	**190 s**	**360 s**	**531 s**	**691 s**	**872 s**
1	6.35	6.76	6.49	6.89	7.27	7.54	7.13
2	7.03	6.25	7.03	7.27	7.41	7.54	7.81
3	6.73	6.87	7.4	7.54	7.78	8.18	8.19
4	6.25	6.25	7.14	7.68	7.95	7.92	8.22
5	6.89	6.76	7.13	7.4	7.54	7.81	8.4
6	6.63	6.72	6.76	7.27	7.78	7.91	8.14
7	7.01	7.17	7.03	7.41	7.13	6.99	7.46
8	7.16	6.76	7.07	7.27	7.17	6.8	6.21
9	6.63	6.86	6.9	6.76	6.62	7.1	6.25

Note: The values were measured from the half tactoid that was unexposed, corresponding to the dynamic map in main Fig. 2h, unit: μm. The No. shows the count of the band for which the half pitch is measured.

Supplementary Table 2 | The dynamics of half pitch values from the original tactoid to two new cholesteric tactoids.

**No.**	**0 s**	**218 s**	**432 s**	**838 s**
1	5.62	5.86	8.14	10.41
2	6.46	6.94	7.4	9.69
3	6.7	7.3	8.14	11.13
4	6.94	6.46	7.42	8.76
5	6.46	6.34	*	*
6	6.58	7.06	*	*
7	6.82	6.34	*	*
8	6.58	6.7	7.54	8.63
9	6.7	7.18	7.3	10.72
10	6.82	6.7	7.18	9.13
11	6.58	6.58	6.34	8.34
12	6.1	5.86	6.7	6.9

Note: The values were measured from the tactoid that was exhibited in Fig. 2l. The No. shows the count of the band for which the half pitch is measured. unit: μm. * no data of half pitch.

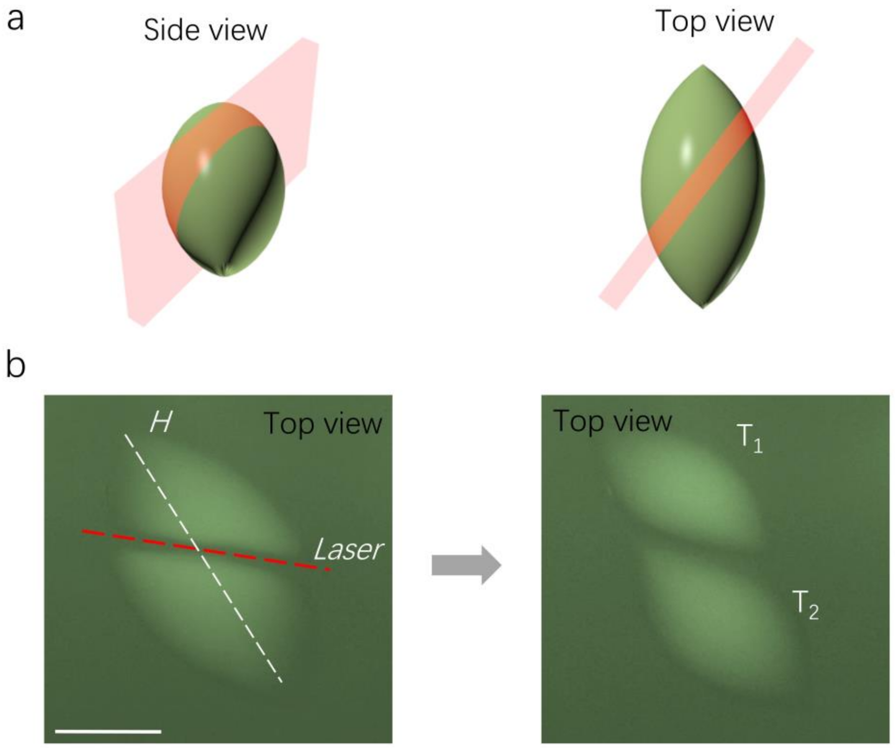

Supplementary Fig. 8 | The break-up of bipolar amyloid tactoids by 3D-Heating. **a**. A schematic of the laser knife that was used to break-up the bipolar tactoid in 45°. The exposed gap was set with 5 μm. **b**. The two separated bipolar tactoids, marked as T_1 (up) and T_2 (down), respectively. Scale bar is 30 μm. All of the images were captured under a crossed polarizer. Models in panel **a** was created with 3ds Max 2021.

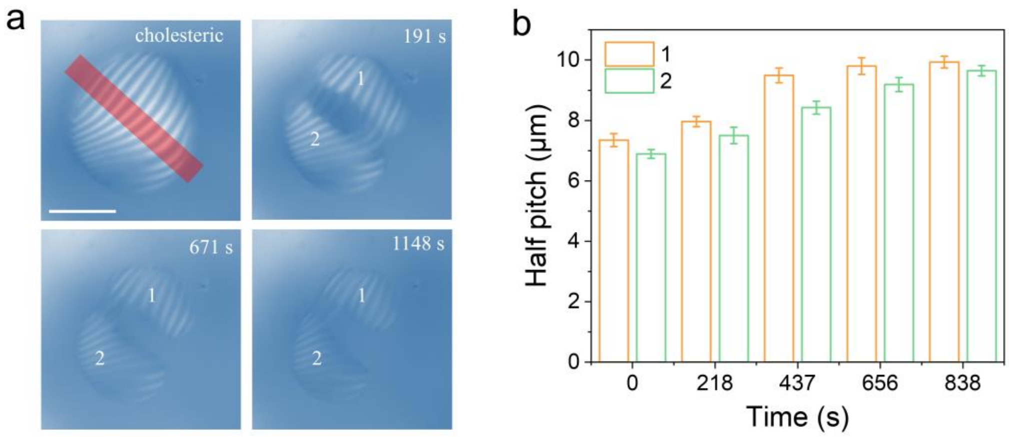

Supplementary Fig. 9 | The break-up of cholesteric amyloid tactoids by 3D-Heating and recovery of tactoid. **a**. The break-up process of a cholesteric tactoid into two cholesteric tactoids (marked as 1 and 2, respectively). The cutting direction was perpendicular to the bands (along *H*), and the exposed gap was set at 15 μm. Scale bar is 50 μm. **b**. The changes in average half pitch values of the two separated cholesteric tactoids measured in the non-irradiation region from **a**. All of the images were captured under a crossed polarizer.

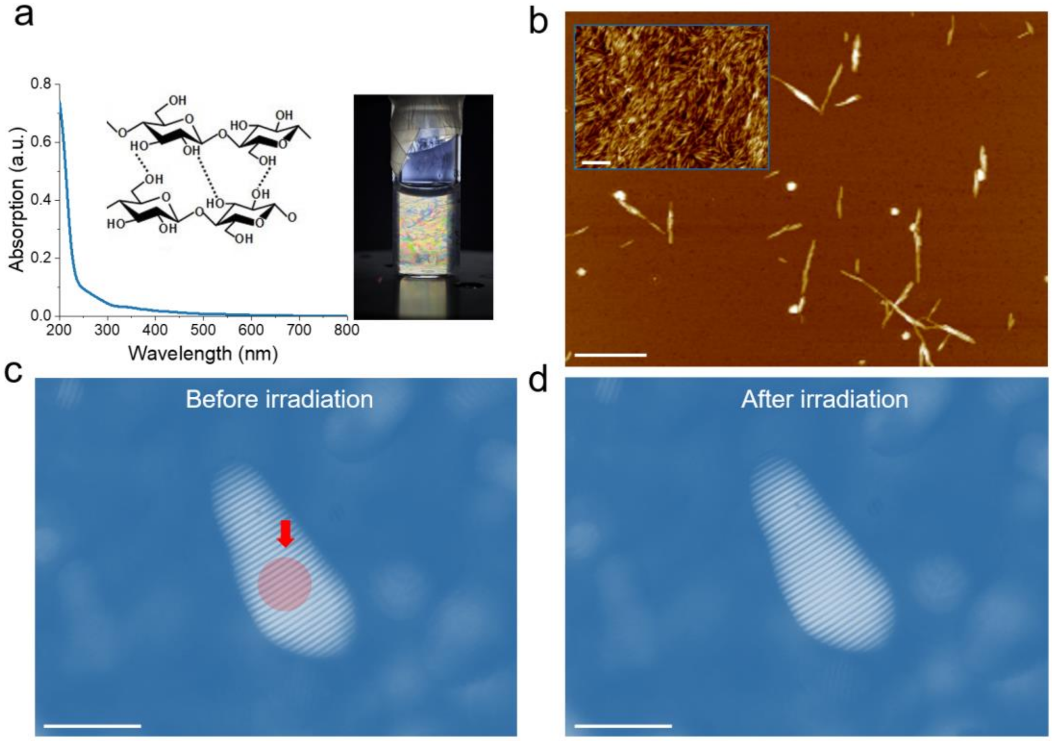

Supplementary Fig. 10 | Exposure of CNCs tactoids. **a**. The molecular structure of CNCs and the UV-VIS spectrum of CNCs suspension. The 4% CNCs suspension shows the formation of birefringent domains. **b**. AFM images of CNCs (diluted ×500). The inset image is the CNCs film on Mica. The scale bar is 500 nm. **c**. POM image of CNCs cholesteric tactoids after an incubation of 4 days. The red region is prepared for the laser exposure. POM image was captured under a crossed polarizer. The scale bar is 60 μm. **d**. POM image checked immediately after the exposure. No visible changes occur in the cholesteric structure exposed in the marked region. The scale bar is 60 μm.

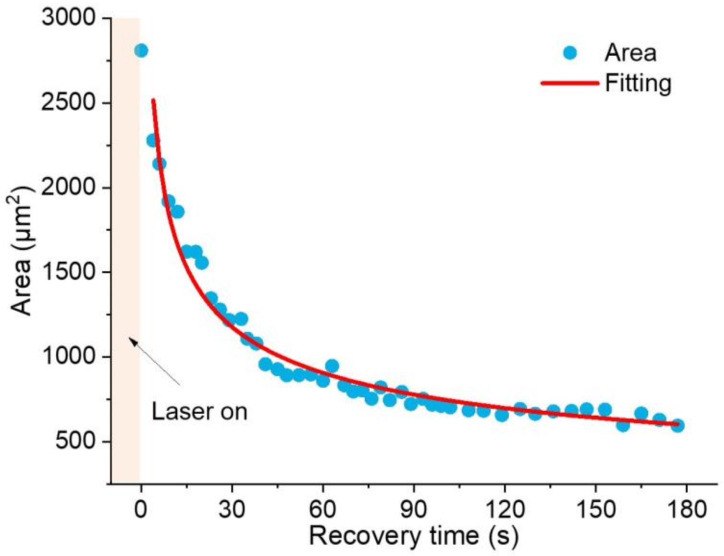

Supplementary Fig. 11 | The recovery rate of the disordered area. A thin disk exposure region (red color) with a thickness of 5 μm and a radius of 30 μm was created in the center of a cholesteric tactoid. The curve shows the recovery of the exposure area that appeared as a shadow under a cross polarizer. The red curve represents the fitting result.

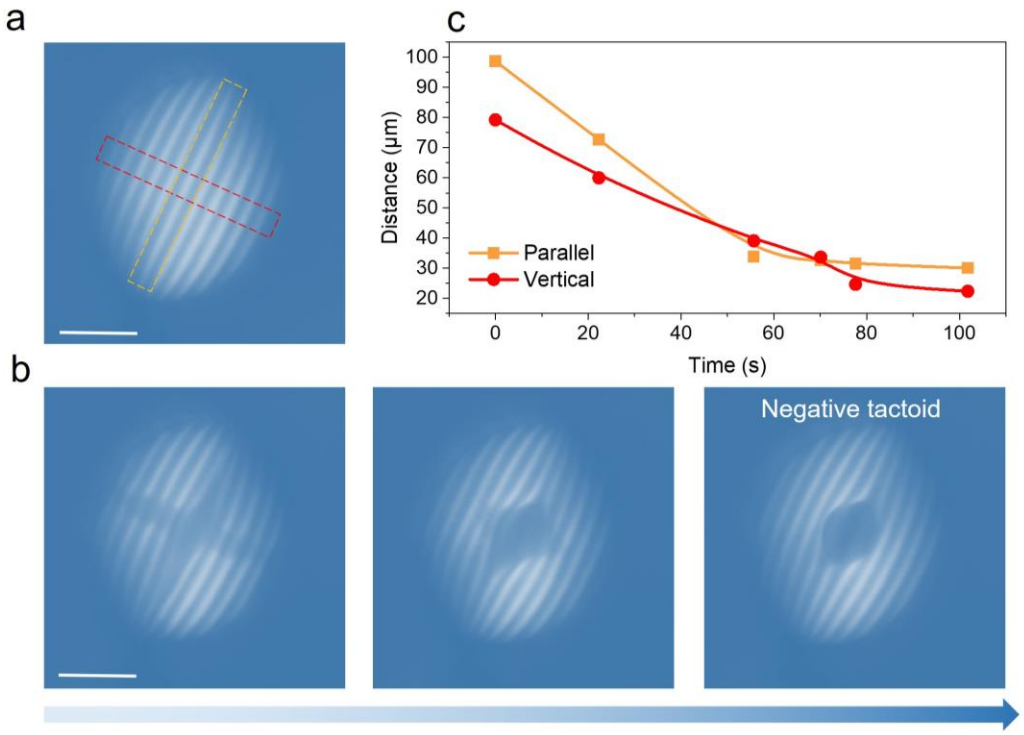

Supplementary Fig. 12 | The exposure of cholesteric tactoids with patterns. **a**. Cross-shaped exposure areas marked in cholesteric tactoids. The length of the longer (red color) exposure isotropic region was 100 μm and the shorter (orange color) one was 80 μm. Scale bar is 30 μm. **b**. A laser was applied following the designed pattern. The exposed region recovered into a negative tactoid with *H* parallel to the bands. Scale bar is 30 μm. **c**. The changes in the length of two tapes over time. Scale bar is 30 μm. All of the images were obtained by POM under a crossed polarizer.

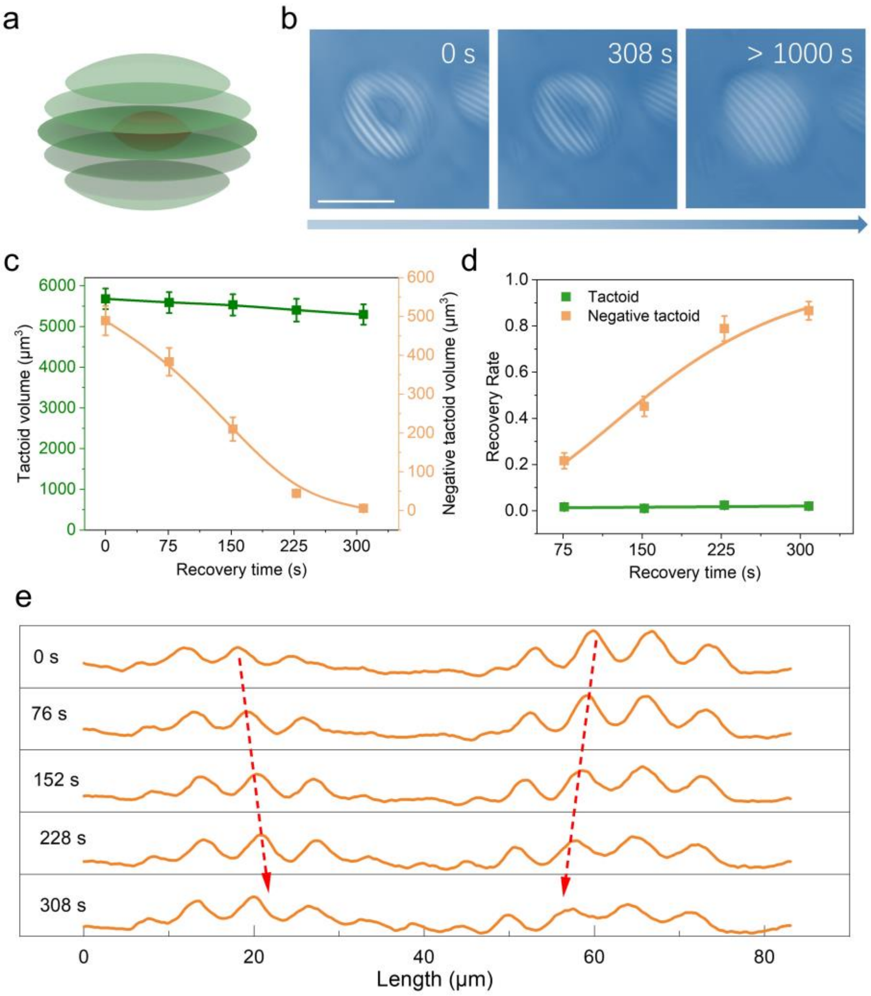

Supplementary Fig. 13 | Laser-induced negative tactoid and its recovery. **a**. Schematic diagram of locally induced negative tactoid in marked position. **b**. The captures of the recovery of the tactoid. The tactoid recovered after >1000 s. The scale bar is 50 μm. All of the images were obtained by POM under a crossed polarizer. **c**. The dynamics of tactoid/negative tactoid volumes vs. the recovery time from **b**. The data were expressed as mean ± standard deviation (S.D). **d**. The evolution of reducing rate of tactoid and negative tactoid from **b**. The data were expressed as mean ± standard deviation (S.D). **e**. The dynamic evolution of half pitch in the recovery of cholesteric tactoid. Models in panel **a** was created with 3ds Max 2021.

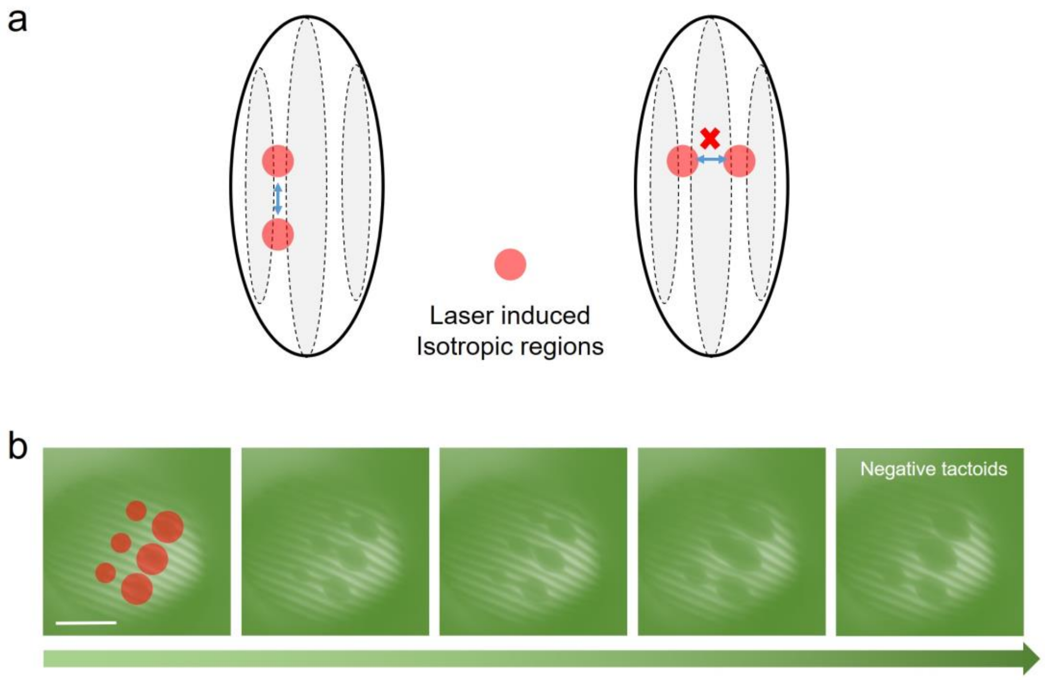

Supplementary Fig. 14 | The exposure of cholesteric tactoids with spots array and their recovery. **a**. Schematic diagram of the exposure areas marked in a single cholesteric tactoid. After the irradiation, the neighbor regions can fuse together along the bands. However, the neighbor unexposed regions between the bands do not fuse. **b**. The dynamic evolution of laser-induced isotropic arrays by POM. The red color represents the exposed areas. The patterns merged together along the bands. The scale bar is 30 µm. All of the images were obtained under a crossed polarizer. Models in panel **a** was created with PowerPoint 2019.

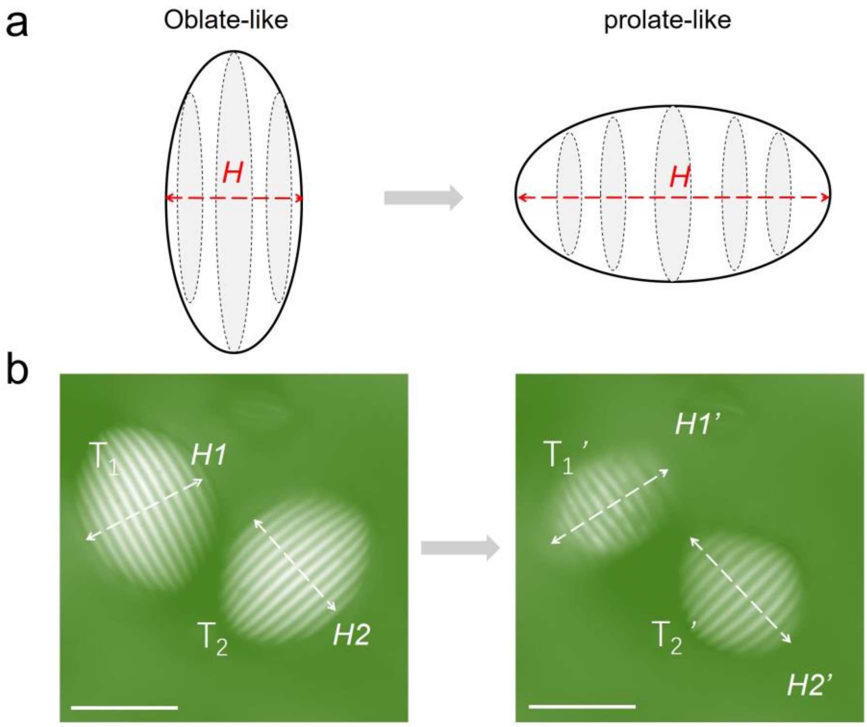

Supplementary Fig. 15 | The erasure of half cholesteric tactoids by 3D-Heating and their recovery. **a**. Schematic diagram of the oblate-like and prolate-like cholesteric tactoid. **b**. The original and finally recovered cholesteric tactoids by POM from Fig. 3j. The scale bar is 50 μm. All of the images were obtained by POM under a crossed polarizer. Models in panel **a** was created with PowerPoint 2019.

Supplementary Table 3 | The dynamics of half pitch values from the remaining half of the cholesteric tactoids. T_1 and T_2 represent the original tactoids, while T_1' and T_2' represent the tactoids after the exposure.

**Item**	**T_1**	**T_1'**	**T_2**	**T_2'**
H (μm)	82	87	79	83
R (μm)	53	31	53	38
Volume (μm^3)	482404.6	175100.9	464755.6	251010.1
Half pitch (μm)	7.0 ± 0.1	7.3 ± 0.1	7.1 ± 0.1	8.4 ± 0.1
Bright bands No.	12	11	11	11

Note: The values were measured from the original tactoids and half tactoids that were unexposed, corresponding to the dynamic map in main Fig. 3j.

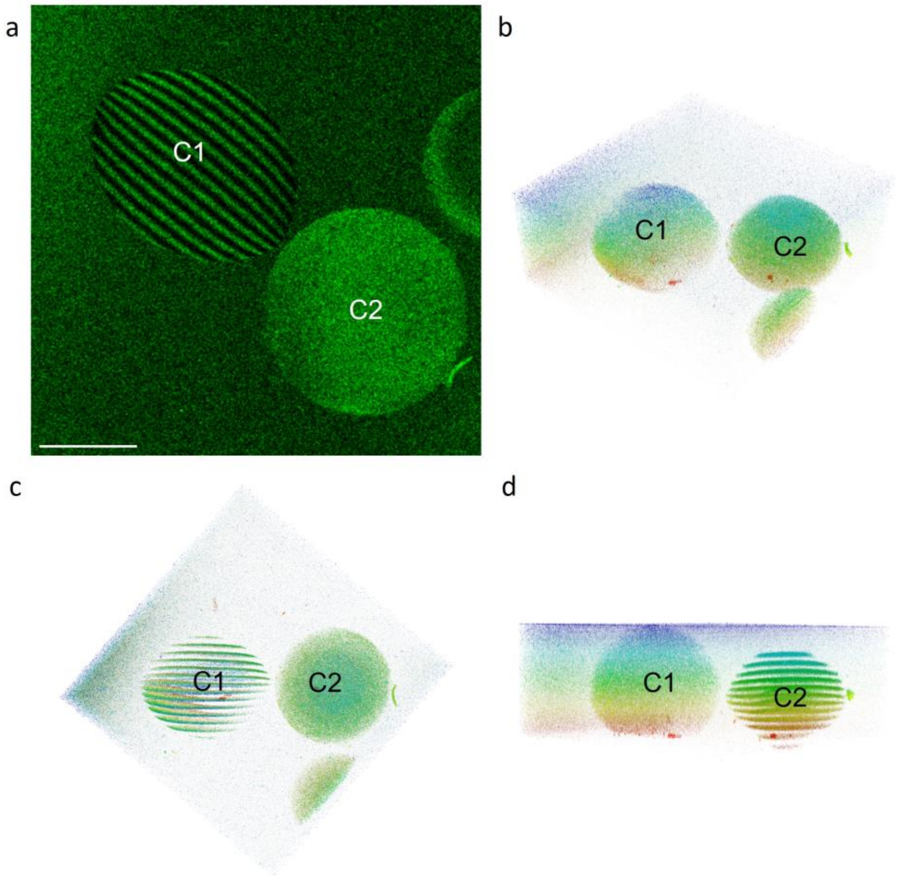

Supplementary Fig. 16 | 3D view of nanoparticle hybrid tactoids by fluorescence microscope. **a**. Confocal fluorescence microscope image of two cholesteric tactoids (named C1 and C2, respectively). The scale bar is 40 μm. **b**. 3D view of fluorescence image of two cholesteric tactoids, from a direction such that no bright bands can be obtained. **c-d**. Top view and side view of the two cholesteric tactoids captured by confocal fluorescence microscope. Obtaining images at different views allows us to distinguish the orientations of the cholesteric tactoids clearly by the bright and dark bands.

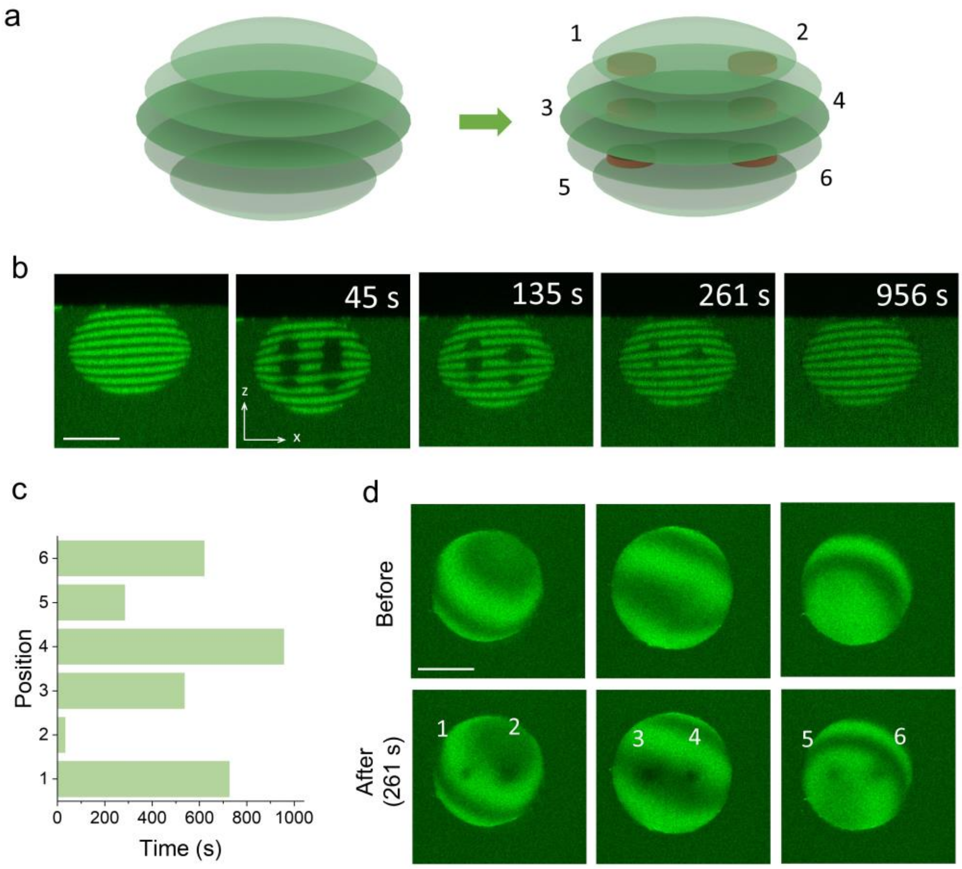

Supplementary Fig. 17 | Laser digging arrays on a single nanoparticle hybrid tactoid and its recovery. **a**. Schematic showing the target locations. **b**. Confocal fluorescence microscope image of a cholesteric tactoid captured in the side view. The spatially distributed six points induced by 3D-Heating and the recovery process. The scale bar is 40 μm. **c**. The disappearing time of each laser-induced isotropic region. As the No. 2 defect merged with No. 4, we marked the No. 2 defect with 0 s. **d**. Top view of captured isotropic regions at 261 s. The upper row exhibits the original states before irradiation. The scale bar is 40 μm. Models in panel **a** was created with 3ds Max 2021.

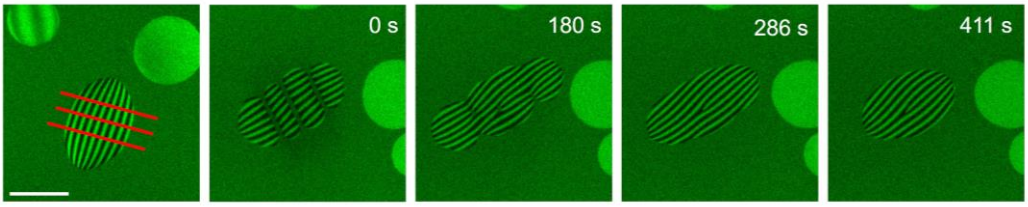

Supplementary Fig. 18 | Cutting of a tactoid into 4 pieces and its quick recovery processes. Three “laser knives” (marked with red color) across the bands (along *H*) in parallel. The broken tactoid recovered in 411 s, with only one mismatched band. The fluorescence images were obtained from the top view of tactoid. The scale bar is 30 μm.

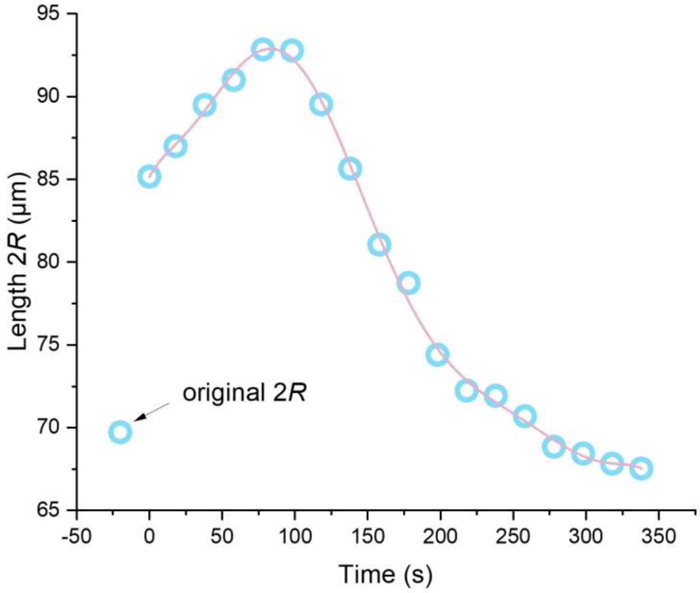

Supplementary Fig. 19 | The dynamics of recovery processes of the three pieces of a cholesteric tactoid. The changes of length (2*R*) in three pieces cholesteric tactoid, as shown in Fig. 4f. The first point represents the original state of 2*R*. The curves were fitted by polynomial.

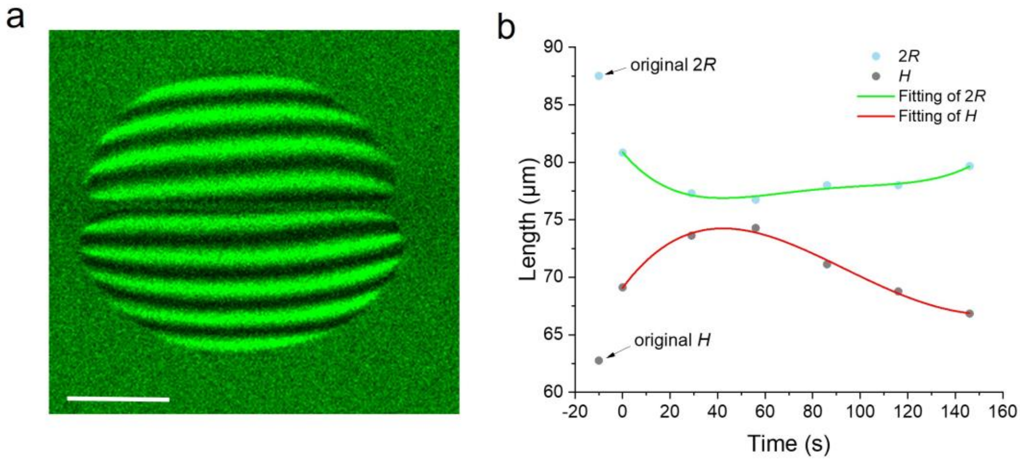

Supplementary Fig. 20. | Cutting of a tactoid along 2*R* and its recovery process. **a**. High resolution image of the separated tactoid in Fig. 4h. The scale bar is 25 μm. **b**. The dynamics of 2*R* and *H* of the separated tactoid in Fig. 4h. The curves were fitted by polynomial.

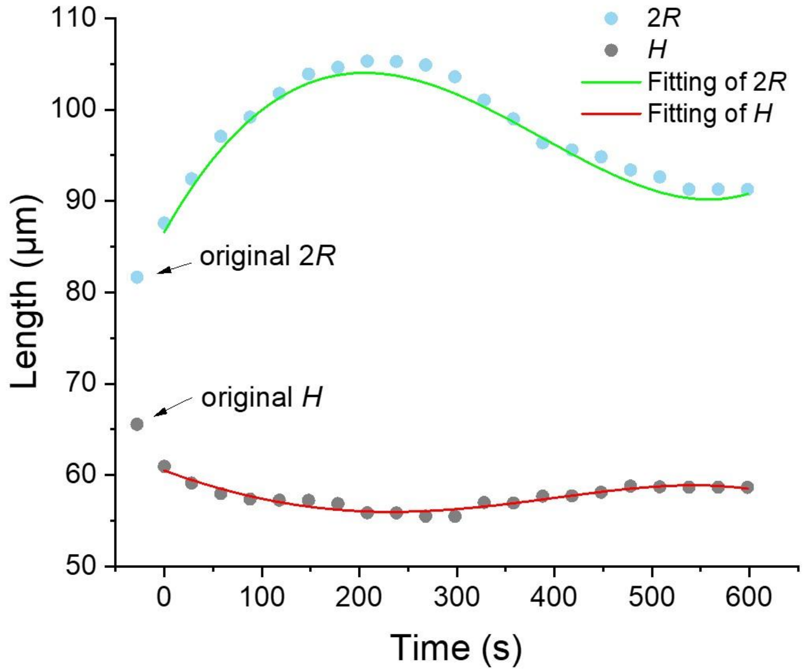

Supplementary Fig. 21 | The dynamics of 2*R* and *H* of separated tactoid along *H*. The dynamics of 2*R* and *H* of separated tactoid in main Fig. 4h, along the *H* direction. The curves were fitted by polynomials.

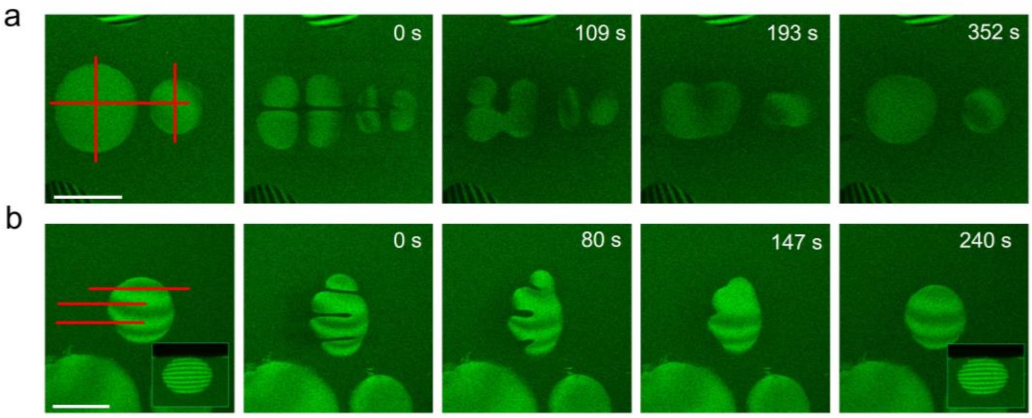

Supplementary Fig. 22 | Random cutting of a tactoid into pieces and its recovery processes. **a** and **b**. The tactoid was cut randomly to a heteromorphosis, but recovered to its original morphology. The inset image shows the side view (*xz* scanning) of the tactoid before/after laser cutting. The dark areas were the glass and air. The scale bar is 30 μm.

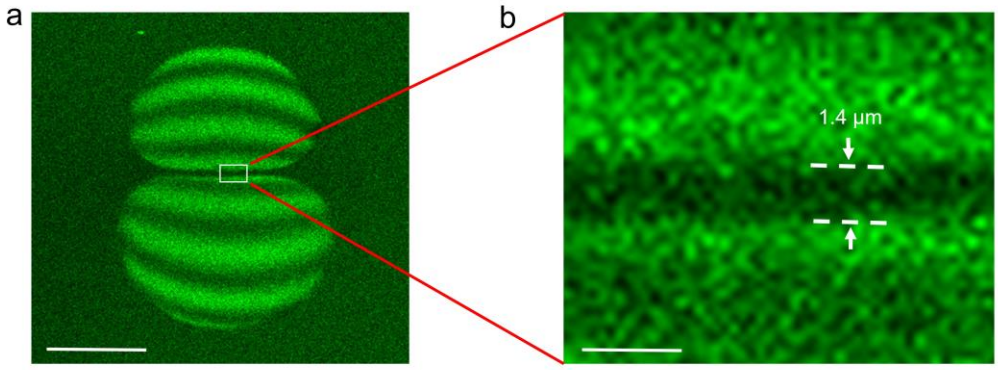

Supplementary Fig. 23 | High resolution cutting by 3D-Heating. **a**. A cholesteric tactoid was cut into two pieces along the 2*R* direction. The scale bar is 30 µm. **b**. Zoomed image of the cutting in (**a**). The cutting gap only reached 1.4 µm. The scale bar is 2 µm.

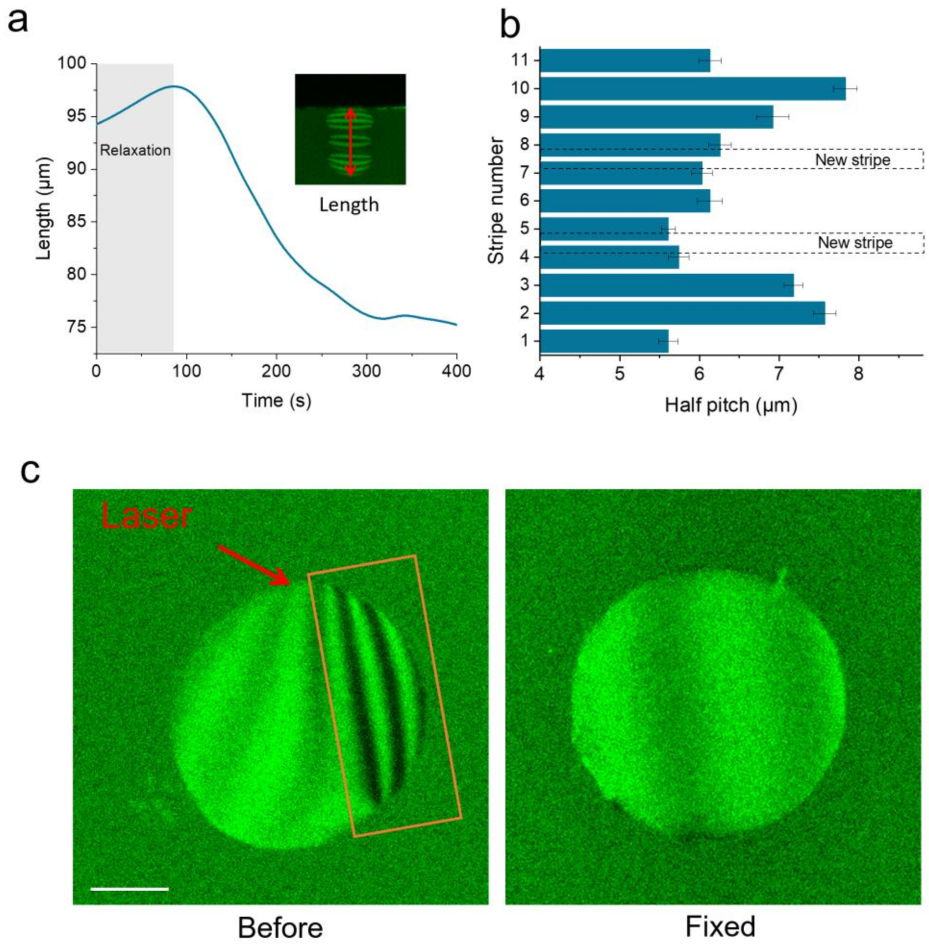

Supplementary Fig. 24 | Engineering of cholesteric tactoid. **a**. The dynamics of tactoid length (H) after irradiation. **b**. The record of half pitch after the irradiation induced two new bands in their expanded region. **c**. The fixing of a tactoid by *3D-Heating*. A two-phase (heterogeneous) tactoid was exposed under the laser on the marked region. The exposure energy was ~0.2 nJ per pulse. With 9 s scanning, the two-phase tactoid annealed into a single-phase tactoid. The scale bar is 30 μm.

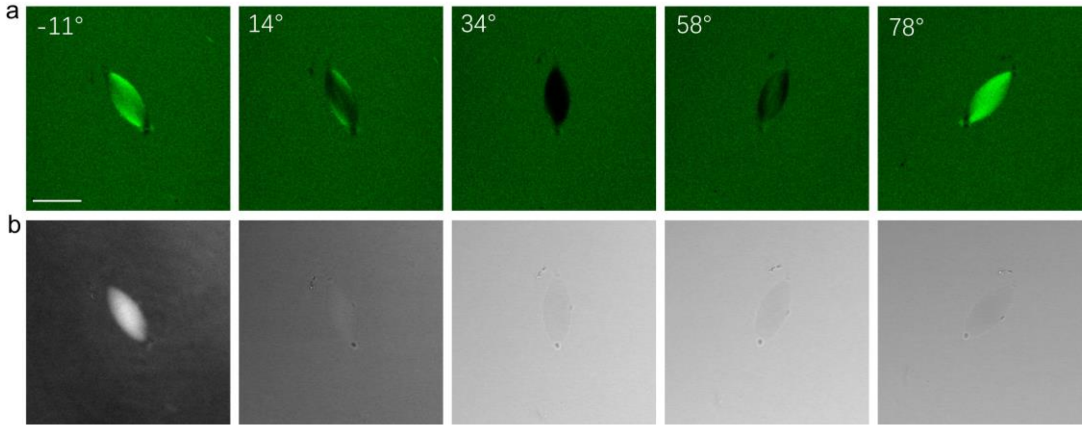

Supplementary Fig. 25 | The imaging of a rotated bipolar tactoid. **a**. The confocal fluorescence image of the tactoid in different rotations. The scale bar is 30 μm. **b**. The corresponding POM images. The background of the POM images changes with the rotation of the sample, while the confocal imaging leaves unaltered background.

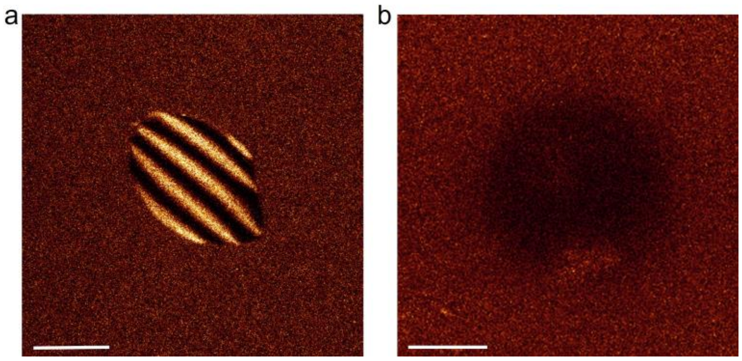

Supplementary Fig. 26 | Erasing a tactoid layer for smart optical switch. **a**. The original image of the tactoid layer. The scale bar is 20 μm. **b**. The tactoid layer was destroyed after irradiation. The scale bar is 15 μm.

References

1 Usov, I. & Mezzenga, R. FiberApp: An Open-Source Software for Tracking and Analyzing Polymers, Filaments, Biomacromolecules, and Fibrous Objects. *Macromolecules* **48**, 1269-1280 (2015).

2 Applegate, M. B., Marelli, B., Kaplan, D. L. & Omenetto, F. G. Determination of multiphoton absorption of silk fibroin using the Z-scan technique. *Opt. Express* **21**, 29637-29642 (2013).

3 Gu, B. *et al.* Z-scan theory for material with two- and three-photon absorption. *Opt. Express* **13**, 9230-9234 (2005).

4 Hanczyc, P., Samoc, M. & Norden, B. Multiphoton absorption in amyloid protein fibres. *Nature Photonics* **7**, 969-972 (2013).

5 Good, M. C., Vahey, M. D., Skandarajah, A., Fletcher, D. A. & Heald, R. Cytoplasmic Volume Modulates Spindle Size During Embryogenesis. *Science* **342**, 856-860 (2013).
