# Supplementary material for: Remodeling and self-healing of individual amyloid tactoids via multiphoton absorption
Source: Nat Commun. 2025 Dec 2;17:254. doi: 10.1038/s41467-025-66954-8 (PMC12783262; doi:10.1038/s41467-025-66954-8)
Supplement: Supplementary file 2 — Description of Additional Supplementary Files [file 41467_2025_66954_MOESM2_ESM.pdf]

Captions for Movies 1-9

Supplementary Movie 1 | The post-dynamic rearrangement of the separated bipolar tactoids by 3D-Heating. The dynamics of the bipolar tactoid that was cut into two parts. The two separated parts are rearranged into two individual bipolar tactoids. The Movie was obtained by POM under a crossed polarizer. The scale bar is 15 μm.

Supplementary Movie 2 | The recovery of a localized isotropic region in a tactoid. The recovery of an isotropic region within a cholesteric tactoid that was created by exposing a selected area to 3D-Heating. The isotropic region relaxes to a negative bipolar tactoid. The Movie was obtained by POM under a crossed polarizer. The scale bar is 25 μm.

Supplementary Movie 3 | The cholesteric nucleation during the recovery of the tactoid. A volume, with 50 μm in diameter, depth reached ~10 μm, within a cholesteric tactoid was exposed to 3D-Heating. The Movie shows that, within the exposed isotropic region, a cholesteric region forms at 142 s. The large isotropic region ultimately recovers its original cholesteric phase. The Movie was obtained by POM under a crossed polarizer. The scale bar is 7.5 μm.

Supplementary Movie 4 | 3D modeling of the cholesteric tactoid. The 3D modeling of a hybrid cholesteric tactoid, which is captured by confocal fluorescence microscopy with 1 μm layer distancing in z-stacking mode. The scale bar is 50 μm.

Supplementary Movie 5 | The recovery of tactoid (cut into three pieces). The hybrid cholesteric tactoid was cut into three parts. The Movie shows the recovery of the three pieces into the original uniaxial cholesteric tactoid. The scale bar is 10 μm.

Supplementary Movie 6 | The recovery of the tactoid (cut into four pieces). The hybrid cholesteric tactoid was cut into four parts. The Movie shows the recovery of the four pieces into the original uniaxial cholesteric tactoid. The scale bar is 25 μm.

Supplementary Movie 7 | The recovery of the tactoid (cut along the *2R*). The recovery of a hybrid cholesteric tactoid, that was cut into two parts along the $2R$ of the tactoid, to the original uniaxial cholesteric tactoid within approximately 2 min. The scale bar is25 μm.

Supplementary Movie 8 | The recovery of tactoid (cut along the *H*). The recovery of a hybrid cholesteric tactoid, that was cut into two parts along the H of the tactoid, to the original uniaxial cholesteric tactoid within approximately 8 min. The scale bar is 25 μm.

Supplementary Movie 9 | Pitch-tunable amyloid tactoids by 3D-Heating. The structural organization of a hybrid cholesteric tactoid is manipulated by 3D-Heating. A 10-band cholesteric tactoid was rebuilt into a 12-band tactoid. The irradiation (with a laser power ~0.2 nJ per pulse) could first trigger the melting of the local order and then induce a new cholesteric band in the exposed region. The half pitch length expanded under exposure, and new periods were induced. The scale bar is 10 μm.
